# The response to substandard and falsified medical products in francophone sub-Saharan African countries: weaknesses and opportunities

**DOI:** 10.1186/s40545-023-00628-y

**Published:** 2023-10-06

**Authors:** Cécile Macé, Jean-Baptiste Nikiema, Omar Serigne Sarr, Patient Ciza Hamuli, Roland Djang’eing’a Marini, Richard Cizungu Neci, Pernette Bourdillon Esteve, Raffaella Ravinetto

**Affiliations:** 1 Independent Consultant, Nantes, France; 2 World Health Organization Regional Office for Africa, Brazzaville, Congo; 3 University of Dakar Cheikh Anta Diop, Dakar, Senegal; 4 Senegalese Drug Regulatory Agency, Dakar, Senegal; 5 Faculty of Pharmaceutical Sciences, LACOMEDA, University of Kinshasa, Kinshasa, Democratic Republic of Congo; 6 CIRM, VibraSante Hub, Department of Pharmacy, Laboratory of Pharmaceutical Analytical Chemistry, University of Liege, Liege, Belgium; 7 Ecumenical Pharmaceutical Network (EPN), Nairobi, Kenya; 8 Incidents and Substandard/Falsified Medical Products Team, World Health Organization (WHO), Geneva, Switzerland; 9 Department of Public Health, Institute of Tropical Medicine, 2000 Antwerp, Belgium; 10 School of Public Health, University of the Western Cape, Cape Town, South Africa

**Keywords:** Medicines, Drugs, Medical products, Substandard, Falsified, Regulation, Pharmacovigilance, Surveillance, Africa

## Abstract

Assuring the quality of medical products manufactured, imported or distributed in francophone sub-Saharan Africa remains a challenge, despite positive signals like the growing engagement in the benchmarking of regulatory authorities and -particularly- in the establishment of the African Medicines Agency. In this short report, we describe the existing activities to prevent, detect and respond to substandard and falsified products (SF) in this region, either through African multilateral organizations and initiatives led by the World Health Organization, or through the contribution of other stakeholders, such as local universities and procurement agencies. We underline that these emerging local stakeholders may play a pivotal role to guide and inform the national regulatory authorities about the prevalence and patterns of SF medical products, complementing the market surveillance and control, and building awareness of the importance of pharmaceutical quality assurance for public health.

## Introduction

Substandard and falsified products (SF) [1, 2] cause morbidity and mortality, fuel resistances [3] and hamper the performance of health systems [4]. Data from Francophone sub-Saharan Africa are limited, but surveys and alerts increasingly suggest that SF medical products are widespread in the region, (non-exclusively) due to the limited capacity of National Regulatory Authorities (NRAs) to fulfil their functions. In this short report, inspired by a workshop held on 1st December 2022, we describe some experiences that could be expanded or replicated for strengthening the awareness of, and the response to SF medical products in this region.

## Multilateral African organizations

Pharmaceutical quality assurance (QA) is high on the agenda of continental organizations, with important aspirational objectives. First, the *African Union* (AU) and the World Health Organization (WHO) started in 2009 the African Medicine Regulatory Harmonization (AMRH) Initiative; and in 2021, following the Treaty ratification by 15 member states, they started the establishment of the African Medicines Agency (AMA). Second, the *Economic Community of West African States* (ECOWAS) endorsed the West African Health Organization (WAHO) Strategic Plan 2016–2020 [5], including plans to foster access to quality-assured essential health products through awareness-raising, regulation and supply. It also adopted the WAHO Strategic Plan “Vision 2030”, including ways to accelerate access to affordable quality health services [6]. Some ECOWAS Member States joined the African Medicines Quality Forum (AMQF-TC) [7], a Technical Committee on strengthening quality control (QC) systems. The ECOWAS Political Declaration on Prevention of Drug Abuse, Illicit Drug Trafficking and Organised Crime in West Africa [8] prompted border surveillance on SF medical products in Côte d'Ivoire, Senegal, and Guinea. Third, the *West African Economic and Monetary Union* [9] harmonised the requirements for marketing authorization, and supports regulatory strengthening based on the AU Model Law [10]. Finally, Benin, Burkina Faso, Guinea, Niger and Togo ratified the *Medicrime Convention*[11].

## WHO initiatives

The *Member State Mechanism* was established to tackle SF medical products from a public health approach [12]. It is governed by a Steering Committee, chaired by one member state for two years, rotating through WHO regions. In October 2023, the chairmanship will be for the African region, opening a possibility for Francophone countries to hold the position for the first time. The WHO also promotes the assessment and upgrade of NRAs with the *Global Benchmarking Tool* (GBT) [13]. Currently, no NRAs in French-speaking Africa reached a maturity level 3, i.e., the minimum target of a stable, well-functioning and integrated regulatory system. However, countries such as Burkina Faso, Niger, Ivory Coast and Senegal are actively engaged in this process and could progress rapidly—the presence of a few mature NRAs, and of WHO pre-qualified QC laboratories, would act as catalysts for the region. Finally, the *Global Surveillance and Monitoring System* [14], through a capillary network of regulatory focal points, maintains a global database of SF products, issues alerts, and provides evidence-based trend analysis. Of sixty alerts published to date, 19 concern 56 products identified in eight Francophone African countries (Cameroon, Cote d’Ivoire, Togo, Niger, Burkina Faso, CAR, Chad, Mali)[15]. However, reporting remains low, due to various barriers, including limited awareness, poor detection capacity, inefficient information systems, and concerns over reputational damage.

## Academia

(West) African academics can contribute to the fight against SF medical products by educating future specialists and policymakers. The *University of Senegal Cheikh Anta Diop* and the *University of Douala* in Cameroon are among the first implementers of a curriculum guide on SF medical products, developed by WHO, International Pharmaceutical Federation, and others, targeting undergraduate pharmacy students [16].

Universities can also play a pivotal role to understand the prevalence and patterns of SF medical products. For instance, the *University of Kinshasa (UniKin)* in the Democratic Republic of Congo built this capability by investing in academic partnerships, particularly with the University of Liège, Belgium. The UniKin QC laboratory, LACOMEDA, is accredited by the Ministry of Public Health for QA and QC of Health Products. It conducts high-quality research, based on a four-phases analytical methodology: a rapid field screening; a second screening in the vicinity; full chemical analysis at central level; and standardized findings’ compilation. For the screening, the program uses portable vibrational spectroscopy tools, chosen based on performance, costs and field feasibility, and suitable for post-marketing surveillance and border screening. This model, where academia builds local capacity through international networks and produces rigorous research to guide policy-makers [17–19], could be replicated elsewhere.

## Procurement agencies

Procurement agencies may engage in surveillance. The Ecumenical Pharmaceutical Network (EPN), an independent, non-profit, Christian organisation committed to provide quality-assured pharmaceutical services, started in 2010 the Minilab project [20], in cooperation with the German Institute for Medical Mission, WHO, the Mission for Essential Drugs and Supplies (Kenya) [21], and the University of Tubingen (Germany). Nine out of nineteen members of the EPN Minilab Network are in Francophone Africa. Focal points at EPN partner organizations are trained to perform a visual inspection of incoming medicines [22], followed by simplified disintegration tests and thin-layer chromatography analyses with Minilab™. As EPN partners are frequently located in rural, hard-to-reach areas, their data can integrate the national post-marketing surveillance of NRAs. In addition, the program increases public awareness about pharmaceutical quality, and facilitates the dissemination of information on SF medical products through publications and alerts [23–25]. This is in line with previous calls for awareness and advocacy about the quality and safety of medicines among frontline healthcare providers and the general public, including efforts to translate technical concepts in lay language—that would in turn enhance vigilance and spontaneous reporting [26, 27].

## Conclusion

Preventing, detecting and responding to SF medical products remains a challenge in Francophone sub-Saharan Africa, despite positive signals like the growing engagement in the WHO GBT process and the construction of AMA. The NRAs already engaged in the AMA should prioritize participation in technical committees, e.g., the AMQF-TC for market surveillance and control. Academic institutions and procurement agencies can effectively support surveillance, and they should systematically share their findings with NRAs. All activities should be framed in universal health coverage, as access to essential medicines will eliminate the demand for illegal markets.

## Data Availability

Not applicable.
